# Barriers to primary care access for asylum seekers and refugees in the UK: a systematic review

**DOI:** 10.1093/eurpub/ckac131.509

**Published:** 2022-10-25

**Authors:** S Harrington, S Sornalingam, R Evenden, N Singh, P Paudyal

**Affiliations:** Primary Care and Public Health, Brighton and Sussex Medical School, Brighton, UK

## Abstract

**Background:**

Recent armed conflicts, in addition to the deterioration of humanitarian conditions across the world, has led to the biggest increase in civilian displacement since World War II. Exploration of the barriers and facilitators to primary healthcare access amongst asylum seekers and refugees (ASRs), from the perspective of both service users and service providers, is important for improving policy and practice to ensure more equitable health care.

**Methods:**

Systematic searches of PubMed, EMBASE, MEDLINE, and CINHL databases were conducted to identify articles until May 2021 using a combination of relevant search terms. Studies were eligible if they were published in English and conducted in ASR populations in primary care settings the UK using qualitative approaches. Literature was thematically analysed using Braun and Clarke’s 6-step process. Quality assessment of included studies was conducted using the Mixed Methods Appraisal Tool.

**Results:**

Nine studies were included in the review. Key themes identified included: accommodation within services; awareness of service navigation, negotiation and eligibility of care; accessibility; availability of appointments; acceptance; complexity within health needs; and cultural appropriateness. Healthcare professionals encounter barriers and facilitators within the healthcare setting, the overall healthcare system, and with regards to their understanding of migration policy. ASRs experience barriers and facilitators with regards to accessing and understanding the health system, cultural appropriateness of care, cost, stigma and prejudice, and availability of specialist services.

**Conclusions:**

This study highlights the difficulties ASRs and healthcare professionals face in primary care settings, the need for consistent and unambiguous guidance that supports the cultural competence of the heath service, and the need for further research into the efforts made to eliminate health discrimination within primary care.

**Key messages:**

• Barriers exist to primary healthcare amongst asylum seekers and refugees.

• Specialised and incorporated healthcare and support is needed due to unique social, cultural, and demographical differences of this population.

